# Large-scale identification of human protein function using topological features of interaction network

**DOI:** 10.1038/srep37179

**Published:** 2016-11-16

**Authors:** Zhanchao Li, Zhiqing Liu, Wenqian Zhong, Menghua Huang, Na Wu, Yun Xie, Zong Dai, Xiaoyong Zou

**Affiliations:** 1 School of Chemistry and Chemical Engineering, Guangdong Pharmaceutical University, Guangzhou, 510006, People’s Republic of China; 2 School of Chemistry and Chemical Engineering, Sun Yat-Sen University, Guangzhou, 510275, People’s Republic of China; 3 SYSU-CMU Shunde International Joint Research Institute, Shunde, 528300, People’s Republic of China

## Abstract

The annotation of protein function is a vital step to elucidate the essence of life at a molecular level, and it is also meritorious in biomedical and pharmaceutical industry. Developments of sequencing technology result in constant expansion of the gap between the number of the known sequences and their functions. Therefore, it is indispensable to develop a computational method for the annotation of protein function. Herein, a novel method is proposed to identify protein function based on the weighted human protein-protein interaction network and graph theory. The network topology features with local and global information are presented to characterise proteins. The minimum redundancy maximum relevance algorithm is used to select 227 optimized feature subsets and support vector machine technique is utilized to build the prediction models. The performance of current method is assessed through 10-fold cross-validation test, and the range of accuracies is from 67.63% to 100%. Comparing with other annotation methods, the proposed way possesses a 50% improvement in the predictive accuracy. Generally, such network topology features provide insights into the relationship between protein functions and network architectures. The source code of Matlab is freely available on request from the authors.

Protein can carry out various biological functions, such as replication and transcription of DNA, catalysis of metabolic reactions, and transport of bioactivity molecules. The accurate annotation of protein function is therefore a vital step to elucidate the nature of life at the molecular level and has a great application in biomedical and pharmaceutical industry^1^,^2^. With the development of high-throughput sequencing technology, an enormous amount of protein sequence has been generated and reserved in database. However, the functionally characterised fraction by various biological experiments is limited owing to the time consuming, expensive and technically challenging^3^,^4^. As of April 2016, the UniProtKB^5^ contains 151,869 human protein sequence entries, of which 20,199 (<15%) entries have been manually annotated. Therefore, it is urgent to develop an automatic and reliable method for the identification of protein function.

At a cellular level, protein executes its function through cooperated interaction rather than autonomous working^6^,^7^. Based on the biological hypotheses that the interacting proteins are more likely to share similar or common functions^8^,^9^,^10^,^11^, many computation methods have been developed to identify protein functions by exploiting protein-protein interaction (PPI) network. These approaches can be roughly divided into three categories: direct annotation, module-assisted and topology method. The first category of direct annotation predicts protein function depending on function information of the direct or indirect neighbors^12^,^13^,^14^,^15^,^16^,^17^,^18^,^19^. Unfortunately, the scheme usually does not consider false positive of PPI^20^, and it also fails when the majority of the neighbors have no function annotations. Unlike inferring function for each protein separately, the second category of module-assisted method firstly identifies the function module and then assigns function to the unannotated protein on the basis of the known function of its member^21^,^22^,^23^,^24^,^25^. Nevertheless, the method usually has high false positive and low prediction accuracy. Moreover, it can only be applied to the protein included in the identified function module. The third category of topology method introduces some new network topology features for characterising proteins, and then predicts protein functions by machine learning algorithm or statistical techniques^26^,^27^,^28^,^29^,^30^,^31^,^32^,^33^. However, the category usually takes no account of PPI false positive and global network topology information.

Over the past several decades, some traditional network topology features, such as degree, betweenness centrality and clustering coefficient, have been developed and utilized to address various biological and medical problems^34^,^35^,^36^. However, these network topology features usually ignore biological or chemical properties of proteins and only treat them as nodes in mathematics. In addition, they only capture topology in the direct vicinity of node under investigation^31^. Therefore, it is still a significant challenge to exploit PPI network and grasp network topology information. A node- and edge-weighted human PPI network is constructed based on primary structure descriptors and interaction confidence scores. Based on the assumption that protein with the same or similar network topology has the same or similar function, the novel network topology features with local and global information are proposed to characterise proteins. Meanwhile, minimum redundancy maximum relevance (mRMR) method is used to select the optimal feature subsets. Finally, support vector machine (SVM) algorithm is employed to construct models for the prediction of protein functions.

## Materials and Methods

### Construction of PPI network

In order to build a high quality human PPI network, PPI data is downloaded from the HIPPIE database^37^ (version 1.6), in which each PPI has an interaction confidence score in the range of [0, 1] for evaluating the reliability of interaction. After removing all self-interactions, duplicate interactions and interactions with confidence score 0, graph theory is utilized to model the information of protein and PPI as an undirected graph. The largest connected component in the graph is the sole consideration, because it contains 97.10% proteins (14,086/14,507) and 90.12% PPIs (153,749/170,604), and can be obtained from the Supplementary Table S1. In the largest connected component, the vertex represents single protein weighted by the protein primary structure descriptor, the edge denotes interaction weighted by the interaction confidence score. The primary structure descriptors include 1767 features, namely, 20 amino acid compositions, 400 dipeptide compositions, 400 normalized Moreau-Broto autocorrelations, 400 Moran autocorrelations, 400 Geary autocorrelations, 21 compositions, 21 transitions and 105 distributions. Compositions, transitions and distributions are some sets of descriptors and developed by Dubchak *et al*.^38^. The amino acid is encoded by the indices of “1”, “2” and “3” based on the attributes such as hydrophobicity, polarity and charges. Compositions have three values, which can be calculated through the number of three indices “1”, “2” and “3” divided by the length *N* of protein sequence. Transitions have three values, which can also be acquired through the number of three combined indices “12”, “13” and “23” divided by the length *N* − 1 of protein sequence. Each specific index class contains 5 distribution values, which are acquired from the position percents in the whole sequence and treated as the first index, 25% index, 50% index, 75% index and 100% index, respectively. The detailed explanations and definitions can be found in reference^38^. All of these features have been widely employed to identify various properties of protein^39^,^40^.

### Collection of protein function

For each protein, the corresponding annotation information of molecular function (MF) and biological process (BP) is retrieved from the UniProtKB/Swiss-Prot (release of July 2014). The function annotation information acquired from reliable Gene Ontology (GO) database^41^ has been conformed manually. Finally, a total of 8474 GO terms are retrieved after excluding the terms marked with evidence codes ISS, ISO, ISA, ISM, IGC, RCA, IEA and NAS.

The selection of negative sample (i.e. protein does not perform a specific function) is especially important for the recognition of protein function. Unlike previous random selection method^3^,^24^, the proteins with GO function term annotations are acquired from the two files of SNOB_human_BP_names.txt and SNOB_human_MF_names.txt in the database of NoGO^42^. Until now, NoGO is the only repertory for negative samples of GO function across the genes of multiple organisms. In the NoGO database, a protein can not perform a specific function if it has the corresponding GO term annotation.

### Construction of protein function dataset

Protein with GO annotation in the UniProtKB/Swiss-Prot is referred to as positive sample, with NoGO annotation as negative sample, without GO annotation in the above two databases as unknown sample. For a given GO term, a benchmark dataset is constructed by the selection of all positive samples and the random selection of negative ones. For those GO terms out of NoGO, the corresponding benchmark datasets are constructed by the choice of all positive samples and the random choice of unknown ones. The proportion of negative or unknown samples to positive ones is set to “1:1”, because it can overcome the limitation of a larger number of negative samples and result in unbiased prediction. In order to guarantee the dataset with statistical significance, the 227 benchmark datasets are finally constructed, in which the number of positive samples is at most 1237 and at least 50. In the corresponding 227 GO function terms, the 45 and 182 terms belong to MF and BP, respectively.

### Characterisation of protein

For each vertex, the breadth-first search algorithm is employed to traverse the component with the change of depth parameter *L (L* = 1, 2, ……, 10). Note that the maximal value of *L* is equal to 10, because the maximal path distance between any two vertexes is 10 (i.e. the distance of protein P51460 and protein Q9NV35 as well as that of protein Q9NV35 and protein Q9UBD3). On the basis of results derived from the breadth-first search, the 8 types of network topology features are then calculated to characterise proteins, and described as follows:

1. The average path vertex weight (*APVW*). For a given vertex *i, APVW* can be obtained by equation (1): 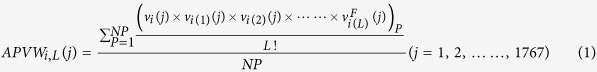 where *v*_*i*_(*j*) and *v*_*i*(*L*)_(*j*) denote the *j*th weight (i.e. *j*th protein primary structure descriptor) of the given vertex *i* and a specific vertex *i*(*L*), respectively. The path distance between the two vertexes is *L*, and *i, i*(1), …, *i*(*L* − 1), *i*(*L*) are involved in the path. The superscript “*F*” in 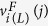 implies that the vertex *i*(*L*) is a protein with a specific function. *NP* is the number of path with distance *L*. For each weight and parameter *L*, a feature value can be obtained. Therefore, the topology feature has 17,670 (1767 × 10) values, which take into account the direct and indirect interaction information between a query protein and the proteins annotated with a specific GO term at the levels of protein primary structure descriptions.
2. The path weight proportion of a protein with a specific function to all proteins (*PWPFP*). The path distance between proteins and the given protein *i* is *L*. The kind of topology features can be calculated by equation (2): 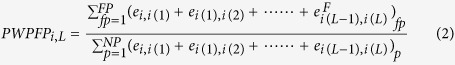
Here *e*_*i*,*i*(1)_ is the edge weight between vertex *i* and *i*(1). The superscript “*F*” in 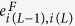 also implies that the vertex *i*(*L*) is a protein with a specific function. *FP* is the number of path with distance *L* and the protein at the end of the path has a specific function. For each parameter *L*, a feature value can be calculated. Therefore, the feature comprises 10 values, which also consider the direct and indirect interaction information between a given protein and the proteins with a specific function at the level of interaction confidence score. The larger value of *PWPFP* means that the query protein can interact with more proteins with a specific function.
3. The average path weight of proteins with a specific function (*APWPF*). The type of topology features can be computed based on the equation (3): 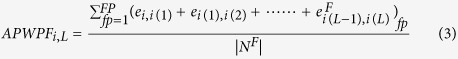
In the equation, *N*^*F*^ and |*N*^*F*^| indicate a set of proteins with a given function and the number of proteins in the set, respectively. The path distance between the proteins and a given protein *i*(*L*) is *L*. By the equation, the 10 features can be obtained when *L* changes from 1 to 10. The network topology features also consider the relationship between a given protein and the proteins with a specific GO term annotation. A higher value of *APWPF* implies that the query protein is more likely to have the specific function.
4. The proportion of interaction number among proteins with a specific function to among all proteins (*PINPFP*). The type of features can be obtained by equation (4): 
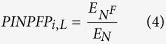
In this equation, *N* is a set of proteins with path distance *L* for the protein *i*. According to the definition, *N*^*F*^ is a subset of *N. E*_*N*_ denotes the number of edges between any two proteins in the set *N*. 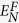 denotes the number of edges between any two proteins in the set *N*^*F*^. Similarly, the 10 values can be obtained based on the various *L*. The topology features take into account the interaction information among direct and indirect neighbor proteins.
5. The average degree value of the proteins with a specific function (*ADPF*). The type of topology features can be calculated by equation (5): 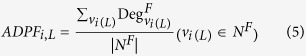 where *Deg ^F^*_*vi*(*L*)_ denotes the degree value of a protein (i.e. vertex *v*_*i*(*L*)_) with a specific function. By changing *L* from 1 to 10, the 10 features can be obtained. A higher feature value of *ADPF* means that the given protein has more interactions with the proteins with a specific function.
6. The average degree and path weight of proteins with a specific function (*ADPWPF*). The type of topology features can be obtained according to equation (6): 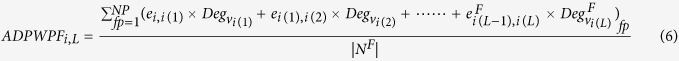
The 10 features can be calculated according to the various *L*, and they consider the interactions with the proteins having a specific GO term annotation.
7. The proportion of protein (*PP*). The type of topology features can be obtained by equation (7): 
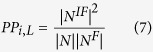
In this equation, *N*^*IF*^ is the intersection of *N* and *N*^*F*^, and |*N*^*IF*^| denotes the number of proteins in the set. Based on the various values of *L*, the 10 descriptor values can also be acquired, and they consider the indirect correlations between the query protein and the proteins with a specific GO term.
8. The proportion of path length (*PPL*). The type of topology features can be calculated by equation (8):
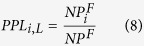
In the equation, 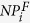 is the number of paths with various distances *L*, in which initial and final vertexes are proteins with a specific function, middle vertexes consist of the given protein *i. NP*^*F*^ is the number of paths, in which the first and the aftermost vertexes are also proteins with a specific function, but medial vertexes do not contain the given protein *i*. In the type of topology features, *L* is equal to 2, 3, 4, ……, 10, because the minimum length of path involving two proteins with a specific function and the given protein *i* is 2. According to the definition, the 9 descriptors can be calculated.

Finally, the feature sets with 8 types of descriptors are employed to characterise proteins. Based on the various *L*, the final feature sets grasp the global and local topology information of proteins. Please refer to Supplement File S1 for a detailed description of these features.

### Construction of model and evaluation of performance

Identification of protein function can be viewed as a problem of two-class classification, i.e., predict a given protein with or without a specific function. Considering the nonlinear relationship between functions and network topology features, therefore, SVM with radial basis function is employed to construct models for the recognition of protein functions. The grid search strategy is utilized to optimise the penalty constant *C* (2^−5^, 2^−3^, ……, 2^15^) and kernel parameter *γ* (2^3^, 2^1^, ……, 2^−15^). The publicly available Libsvm software^43^ is downloaded to predict the protein functions.

The large feature sets can fully characterise the network topology information, but it may result in over-fitting and massive computations. Therefore, mRMR algorithm is employed to select the optimal feature subsets. Based on the mutual information theory, mRMR algorithm ranks features according to their relevance to the class of samples and redundancy among the features. The top ranked features have the better trade-off between maximum relevance and minimum redundancy. The detailed description for mRMR can be obtained from the work of peng^44^ and the algorithm is downloaded from http://penglab.janelia.org/proj/mRMR/index.htm.

Based on the results of 10-fold cross-validation, accuracy (Acc), sensitivity (Sen), specificity (Spe), precision (Pre) and Matthew’s correlation coefficient (Mcc) are calculated to estimate the prediction performance of model. The receiver operating characteristic curve (ROC) is the plot of false positive rate against true positive rate with the various decision thresholds, and precision recall curve (PRC) reflects the relationship between precision and recall with the gradual change of thresholds. The corresponding areas under the curves of ROC (AUCR) and PRC (AUCP) are also calculated to assess the predictive ability. The area is always between 0 and 1, and the model with a higher area value gives a better predictive performance.

## Results and Discussion

### Optimization and evaluation of model

**Figure 1:**
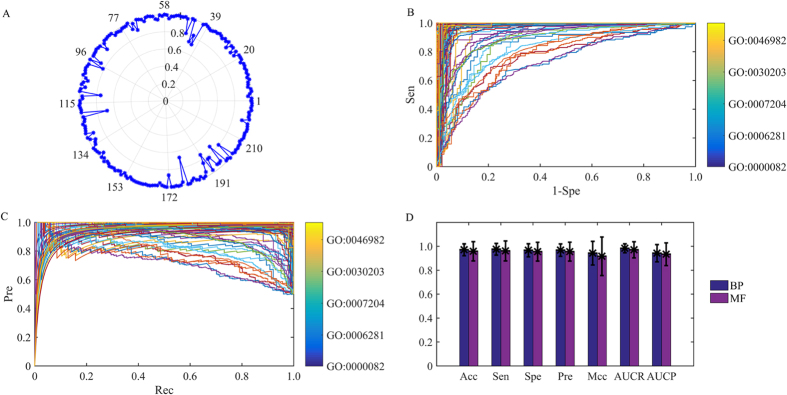
The prediction results of optimized feature subset for 227 benchmark datasets. (**A**) In polar coordinate system, radius is 1 and blue asterisks represent the corresponding Acc values. (**B**,**C**) The receiver operating characteristic and precision recall curves. Each curve has a different color, and changes from blue to yellow. (**D**) The statistical average results for BP (blue) and MF (purple) GO term. The panel indicates the mean value of accuracy, sensitivity, specificity, precision, Matthew’s correlation coefficient as well as area under receiver operating characteristic and precision recall curve. The vertical bar shows the corresponding standard deviation.

For each of 227 benchmark datasets, 200 feature subsets containing 5, 10, 15, ……, 1000 features are generated by mRMR feature selection algorithm. Finally, the 227 optimised feature subsets are obtained based on the strategy of 10-fold cross-validation test and the value of Acc. The corresponding results are shown in Fig. 1 (detailed in Supplementary Table S2).

As shown in Fig. 1(A), the performance of constructed models for most of GO terms are outstanding. The 210 values of Acc, Spe and Pre, and 208 values of Sen are higher than 90%. For Mcc, 194 results are larger than 0.9. The results from Fig. 1(B) and (C) suggest that all values of AUCR and AUCP are higher than 0.7, among them of 215 and 209 values are larger than 0.9. These results reveal that the satisfactory results can be obtained from the optimal feature subsets.

The predictive performance is also investigated for terms from BP and MF. As shown in Fig. 1(D), for all GO terms belonging to BP, the mean values and standard deviations are 97.11% and 4.97% (Acc), 97.54% and 4.95% (Sen), 96.67% and 5.50% (Spe), 96.77% and 5.22% (Pre), 0.9426 and 9.90% (Mcc), 0.9833 and 3.72% (AUCR), 0.9337 and 9.46% (AUCP). For all MF GO terms, the mean values and standard deviations are 95.82% and 8.05% (Acc), 96.14% and 9.34% (Sen), 95.49% and 7.86% (Spe), 95.50% and 7.88% (Pre), 0.9165 and 16.09% (Mcc), 0.9708 and 6.76% (AUCR), 0.9424 and 7.21% (AUCP). In addition, Wilcoxon rank sum test is also performed, and *P* values of 0.6800 (Acc), 0.8257 (Sen), 0.3821 (Spe), 0.3941 (Pre), 0.6744 (Mcc), 0.5534 (AUCR) and 0.9062 (AUCP) are obtained. The results reveal that our method has no significant differences in the predictive performance for BP and MF terms.

### Analysis of the optimal feature subset

**Figure 2:**
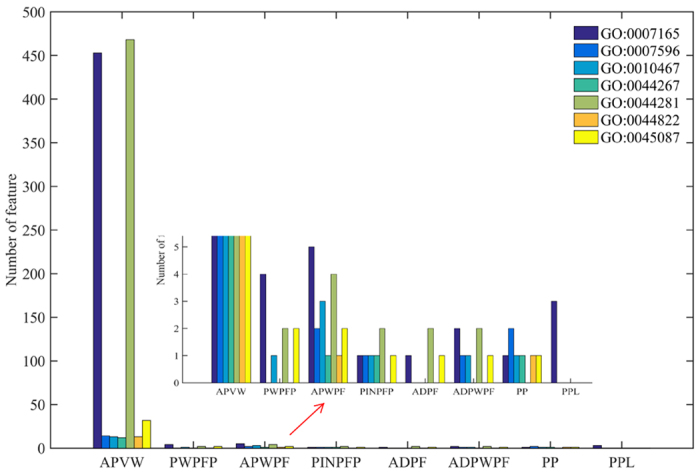
The composition of the optimized feature subset.

In order to avoid tedious work, we only analyse 7 optimal feature subsets derived from the 7 largest benchmark datasets with the corresponding terms GO: 0007165, GO: 0007596, GO: 0010467, GO: 0044267, GO: 0044281, GO: 0044822 and GO: 0045087. The compositions of these best feature subsets are shown in Fig. 2.

From Fig. 2, the optimized feature subset for GO: 0007165 is composed of 470 features, including 453 *APVW* features, 4 *PWPFP* features, 5 *APWPF* features, 1 *PINPFP* feature, 1 *ADPF* feature, 2 *ADPWPF* features, 1 *PP* feature and 3 *PPL* features. In the 453 *APVW* features, 94, 59, 47 and 37 features are derived from the node weight of hydrophobicity, flexibility, polarizability, and charges, all of which are crucial for protein function. Hydrophobicity is an important factor for understanding protein folding and protein function. Flexibility plays a vital role when proteins respond to environmental changes, ligand binding and chemical modifications^45^. Polarizability is a foundation on which various protein primary structure features are developed to identify DNA-binding residues of protein^46^ and G-protein coupled receptors^47^. Surface charges are reported to have more impact on the binding of ion with proteins than general hypothesis^48^ while electrostatic charge at the membrane surface is a crucial determinant of the localization and activation of many proteins^49^.

For GO: 0044281, the optimal feature subset consists of 468 *APVW* features, 2 *PWPFP* features, 4 *APWPF* features, 2 *PINPFP* features, 2 *ADPF* features, and 2 *ADPWPF* features. Among the 468 *APVW* features, 100, 50, 48 and 38 are calculated by node weights of hydrophobicity, polarizability, flexibility and charge. For GO: 0007596, GO: 0010467, GO: 0044267, GO: 0044822 and GO: 0045087, the number of overall features and *APVW* features in the optimized feature subsets are 20 and 14, 20 and 13, 15 and 12, 15 and 13, 40 and 32, respectively. Compared with other features, *APVW* can effectively grasp the network topology information by taking account of physicochemical attributes of direct and indirect neighbor proteins, and it is the dominant one because most of features come from *APVW* (>60%). Except for GO: 0007165, the other 6 corresponding optimised feature subsets have no *PPL* feature, indicating that *PPL* has the least discrimination information. The contributions of *PWPFP, APWPF, PINPFP, ADPF, ADPWPF* and *PP* are roughly equivalent. Therefore, we can expect it is of great significance for predicting protein function if a new encoding strategy can take into account the physical and chemical properties of protein and more effectively consider direct and indirect interaction information in the context of PPI network.

### Effect of random sampling on model performance

**Figure 3:**
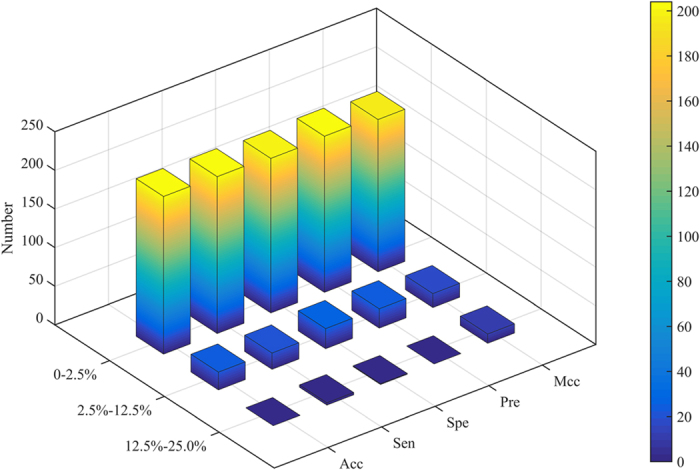
The statistical results of standard deviations.

In the current study, negative or unknown samples with the same size to positive ones are randomly selected to construct benchmark dataset. For each of GO terms, the corresponding 10 datasets through 10 times random sampling are generated to construct predictive models and evaluate their performances by 10-fold cross-validation test. Finally, the standard deviations of Acc, Sen, Spe, Pre and Mcc are calculated to evaluate whether it is rational to sample only once. The statistical results of standard deviations are illustrated in Fig. 3 (detailed in Supplementary Table S3).

For most of GO terms, the corresponding standard deviations are extremely low. For Acc, Sen, Spe, Pre and Mcc, standard deviations of 204, 203, 200, 202 and 197 GO terms consistently fluctuate from 0 to 2.5%, suggesting that the results of 10 duplicated experiments are very close to each other and it is reasonable to perform the random sampling only once. We assume that the standard deviations of GO terms greater than 2.5% are mainly ascribed to the unknown samples involving in some false negative proteins (i.e. these proteins have the corresponding functions, but still have not been recognised). The number of false negative protein in the every benchmark dataset varies widely, resulting in a large deviations of predicted results. However, the problem can be solved through the accumulation and abundance of protein function data.

### Robustness of the current method on sequence similarity

**Figure 4:**
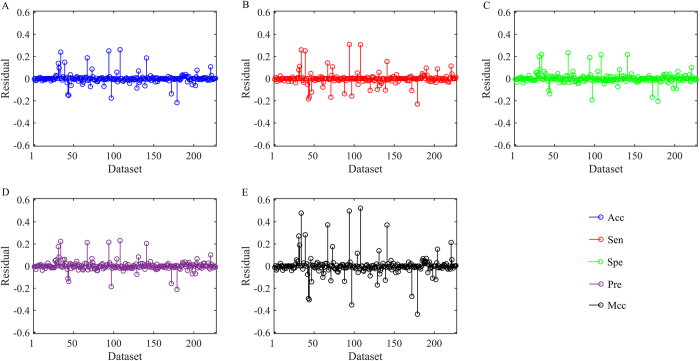
The residual results between benchmark datasets and non-redundant datasets. (**A**–**E**) Represent the residual results of accuracy, sensitivity, specificity, precision and Matthew’s correlation coefficient, respectively. The residual values are indicated by circles terminating each stem.

In order to characterise the network topology information of protein, the 8 kinds of novel features are proposed based on the protein primary structure descriptions. Considering the effect of sequence similarity, proteins in the benchmark datasets with more than 40% sequence similarity are eliminated by CD-HIT software^50^ and thus produce 227 non-redundant datasets. The residual results between benchmark datasets and non-redundant datasets are shown in Fig. 4.

The Acc values of 121 non-redundant datasets are lower than those of benchmark datasets, while the residual values between 108 benchmark datasets and the corresponding non-redundant datasets are lower than 5%. From Fig. 4(B,C,D), 132 residual values of Sen, 118 of Spe and 117 of Pre are lower than 5%. The results indicate that our method is robust for about half of GO terms, and it is still able to maintain prominent predictive performance even if the sequence similarity has decreased to 40%.

Surprisingly, we find from Fig. 4(A) that some residual values are lower than 0, which means that Acc values of non-redundant dataset are higher than those of benchmark dataset. For example, the Acc values of GO: 0042803 from non-redundant dataset (89.35%) are about 22% higher than that from benchmark dataset (67.63%), while the Acc values of GO: 0007173 from non-redundant dataset (99.07%) increase about 18% compared to that from benchmark dataset (81.50%). The results can be explained as follows: (1) By sequence alignment, the amount of proteins in non-redundant dataset is reduced, resulting in the decrease of false negative protein. (2) Sequence alignment leads to positive and negative samples to be identified more easily.

### Effect of degree distribution on model prediction performance

**Figure 5:**
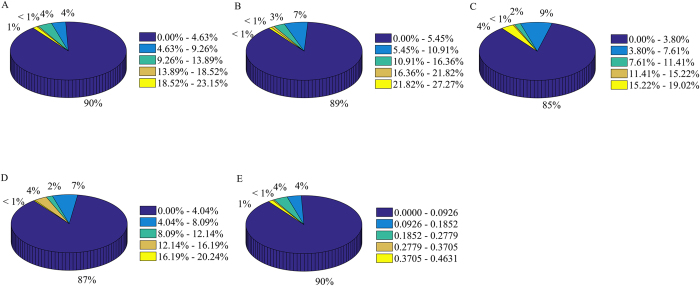
The statistical results of absolute residual values between new datasets containing positive and negative samples or positive and unknown samples with same degree values and benchmark datasets. (**A**–**E**) Indicate the results of accuracy, sensitivity, specificity, precision and Matthew’s correlation coefficient.

In our study, some network topology features are proposed based on the degree value of protein. Therefore, the influence of degree distribution on model prediction performance is further investigated. Firstly, the new datasets are constructed by selecting positive and negative samples or positive and unknown samples according to the same degree values. Secondly, the optimal feature subsets are chosen (the corresponding results shown in the Supplementary Table S4). Finally, the statistical results of the absolute residual values between new datasets and benchmark datasets are calculated and shown in Fig. 5.

The 205 (205/277 = 90%) absolute residual values of Acc are lower than 4.63%, 201 (201/277 = 89%) of Sen lower than 5.45%, 194 (85%) of Spe lower than 3.80%, 198 (87%) of Pre lower than 4.04% and 205 (90%) of Mcc lower than 0.0926. The results reveal that most of absolute residual values are very low and the degree distribution of protein has a minor influence on the prediction results for the vast majority of datasets. It may be attributed to the fact that the optimal feature subsets have very few network topology features of *ADPF* and *ADPWPF*.

About 2% of Acc absolute residual values, 2% of Sen, 5% of Spe, 5% of Pre and 2% of Mcc are higher than 13%, 16%, 11%, 12% and 0.28, respectively. In these datasets, the degree distribution of protein has some impacts on the prediction results, probably resulting from the false negative samples in unknown ones which bring about the unstable prediction performance.

### Comparing with the existing method

The proposed approach is compared with the existing methods, such as diffusion state distance^33^ (DSD), diffusion state distance incorporating confidence^28^ (cDSD), scale-aware topological measures^31^ (STMs), PPI information^29^ (PPIi), network neighbor (NetNei) and sequence similarity (SeqSim). DSD is a new metric based on a graph diffusion property and can capture finger-grained distinctions in proximity for transfer of functional annotation in PPI network. cDSD is an incremental version of DSD and considers the confidence score of PPI. STMs is a scale-invariant description of the topology around or between proteins with a network smoothing operation and diffusion kernels. PPIi considers the function information of neighbor proteins and the weights of interactions, and utilizes a so-called “inclined potential” to infer whether the protein performs a specific function. In SeqSim method, the similarity is calculated based on BLOSUM50 scoring matrix and Needleman-Wunsch algorithm, and then the function of protein with the highest similarity is assigned to a given protein. In NetNei method, the function of a given protein is assigned by counting the occurrence frequency of GO terms of its immediate interacting proteins.

**Figure 6:**
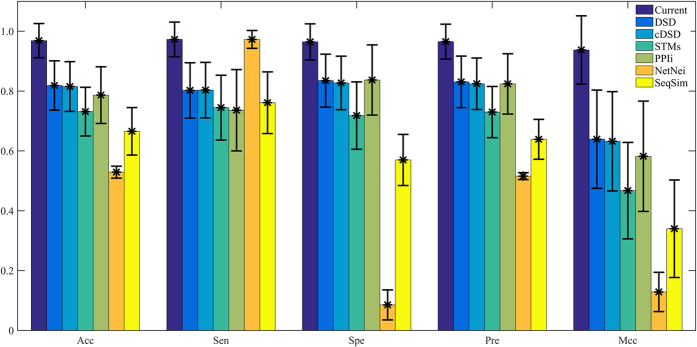
The mean values and standard deviations of results from the current and existing method. The panel shows the mean value of accuracy, sensitivity, specificity, precision and Matthew’s correlation coefficient for our and existing methods. The vertical bar indicates the corresponding standard deviation.

In DSD, cDSD, STMs and PPIi methods, feature selection technique and machine learning method are not employed to identify protein function. In order to compare the proposed method with the existing methods fairly, therefore, we firstly calculate the features, and then use mRMR to select the optimized feature subsets. The selection of optimal feature subsets is not performed for PPIi because only 7 features are calculated according to the definition. Finally, we utilize SVM to construct models and adopt grid search to optimise parameters. The mean results and standard deviations of all GO terms are shown in Fig. 6, and the detailed results can be obtained from the Supplementary Table S5.

From Fig. 6, the current method achieves the highest average values of Acc (96.85%), Sen (97.26%), Spe (96.44%), Pre (96.52%) and Mcc (0.9374). DSD and cDSD obtain the second and third best mean values of Acc (82.86% and 81.52%), Pre (83.07% and 82.44%) and Mcc (0.6391 and 0.6322), respectively. In addition, the two methods acquire the third and fourth largest average values of Sen (80.23% and 80.32%) and Spe (83.49% and 82.72%). STMs, PPIi and SeqSim achieve the low average values of Acc, Pre and Mcc. Although NetNei obtains the average value of Sen 97.26% (the same result with our method), it achieves the lowest values for Acc, Spe, Pre and Mcc at the same time. From these results, we can conclude that the performance of our method is excellent and can correctly identify protein functions.

Wilcoxon rank sum test is also carried out, and the corresponding *P* values are listed in Table 1. Among Acc, Sen, Spe, Pre and Mcc, *P* values are always in the range of [10^−51^, 10^−76^], indicating the significant difference between the current method and existing methods in the prediction performance. Therefore, it is anticipated that our method is superior to these approaches and the proposed network topology features have more useful information in predicting protein function.

### Large-scale identification of protein function

In order to demonstrate the practical application of the proposed method, we perform predictions of 7 GO terms for negative and unknown samples. The results are listed in Table 2. For GO terms of 0044281, 0007165, 0010467, 0045087, 0044267 and 0007596. The corresponding values of Spe are 74.26% (7,301/9,832), 87.02% (7,766/8,924), 90.75% (7,987/8,801), 83.19% (10,111/12,154), 83.13% (7,986/9,844) and 94.04% (11,722/12,465), respectively. These results are very close to those listed in Supplement Table S2, indicating an outstanding generalization performance for the constructed model.

For GO term of 0044281, 675 proteins with unknown function annotations are predicted with the function of small molecule metabolic process, and some of them have indirect evidence supports. For example, we find that 105 proteins have the function annotation with ancestor or descendant based on the GO database, implying that these proteins perform the functions related to the biological function and/or biological process. In addition, we also find that the 338 proteins have the neighbor proteins with the annotation, suggesting that these proteins are likely to have the function because the interacted proteins generally have similar or common functions. For GO terms of 0007165, 0010467, 0045087, 0044267 and 0007596, the 1,005, 684, 154, 656 and 77 proteins originally annotated as unknown functions are predicted to have the corresponding function of GO terms. In these predicted proteins, 250 (24.88%), 60 (8.92%), 17 (11.04%), 83 (12.65%) and 3 (3.90%) proteins have the corresponding GO terms with ancestors or descendants. Also, 529 (52.64%), 319 (46.64%), 83 (53.90%), 324 (49.39%) and 35 (45.45%) proteins have the neighbor proteins with the annotation of the corresponding GO terms. These results indicate that most of the predicted proteins have indirect evidences from GO annotations and human PPI network, and the predicted results are very reliable.

## Conclusion

The identification of protein function is an important step for deciphering the nature of life. In this study, a novel approach is developed to predict protein functions based on weighted human PPI network and graph theory. The novel network topology features are proposed to characterise proteins in the context of network. The performance of current method is assessed by 10-fold cross-validation test as well as the superiority is proved by comparing with the existing approaches. Also, the robustness of the current approach for redundant data and random sampling is verified by constructing non-redundant datasets and performing duplicated experiments. Our study gives a new insights for protein function mechanism at the scale of PPI network. It is anticipated that the proposed method may be a powerful tool for the annotation of protein function.

## Additional Information

**How to cite this article**: Li, Z. *et al*. Large-scale identification of human protein function using topological features of interaction network. *Sci. Rep.* **6**, 37179; doi: 10.1038/srep37179 (2016).

**Publisher’s note**: Springer Nature remains neutral with regard to jurisdictional claims in published maps and institutional affiliations.

## Supplementary Material

Supplementary Information

Supplementary Information

Supplementary Information

Supplementary Information

Supplementary Information

Supplementary Information

## Figures and Tables

**Table 1:** The *P* values of Wilcoxon rank sum test between the results of current method and the results of existing approaches.

	DSD	cDSD	STMs	PPIi	NetNei	SeqSim
Acc	4.08E-59	5.86E-60	1.46E-69	1.59E-63	5.28E-76	2.16E-73
Sen	1.34E-62	1.55E-62	2.50E-68	2.57E-66	4.51E-07	5.85E-67
Spe	8.18E-51	4.62E-53	7.05E-68	4.19E-51	3.78E-76	1.76E-75
Pre	3.63E-52	3.54E-54	9.87E-69	2.73E-54	3.78E-76	9.82E-75
Mcc	3.87E-59	5.46E-60	1.73E-69	2.62E-63	5.28E-76	3.83E-73

**Table 2:** The Predicted results of the current method for the proteins contained in the constructed protein-protein interaction network.

GO	Negative	Unknown
0007165	7766 (8924)	1005 (3814)
0007596	11722 (12465)	77 (781)
0010467	7987 (8801)	684 (3967)
0044267	7986 (9844)	656 (3298)
0044281	7301 (9832)	661 (1780)
0044822	—	10537 (11948)
0045087	10111 (12154)	154 (920)

Numbers in the second column indicate the numbers of correctly predicted negative proteins and numbers contained in parentheses represent the number of negative proteins. Numbers in the third column mean the number of predicted proteins with the specific function and numbers included in parentheses represent the number of proteins original annotated as unknown function.
