# Supplementary material for: Large-scale identification of human protein function using topological features of interaction network
Source: Sci Rep. 2016 Nov 16;6:37179. doi: 10.1038/srep37179 (PMC5111120; doi:10.1038/srep37179)
Supplement: Supplementary Information [file srep37179-s1.pdf]

Large-scale identification of human protein function using topological features of interaction network

Zhanchao Li[1] *, Zhiqing Liu[1], Wenqian Zhong[1], Menghua Huang[1], Na Wu[3], Yun Xie[1], Zong Dai[3], Xiaoyong Zou[2, 3] *

[1]School of Chemistry and Chemical Engineering, Guangdong Pharmaceutical University, Guangzhou, 510006, People's Republic of China

[2]SYSU-CMU Shunde International Joint Research Institute, Shunde, 528300, People's Republic of China

[3]School of Chemistry and Chemical Engineering, Sun Yat-Sen University, Guangzhou, 510275, People's Republic of China

*Correspondence and requests for materials should be addressed to Z.L. (email: zhanchao@gmail.com) or X.Z. (email: ceszxy@mail.sysu.edu.cn)

A random network with 7 proteins and in Figure S1 is chosen as an example to describe the calculation of *APVW*, *PWPFP*, *APWPF*, *PINPFP*, *ADPF*, *ADPWPF* and *PP*. In the network, the path distance between A and B, A and C as well as A and D is 1. The path distance between A and E, A and F as well as A and G is 2, and the corresponding path is A-B-E, A-B-F and A-C-G. We assume that interaction confidence score between A and B, A and C, A and D, B and C, B and D, B and E, B and F, C and G, C and D, E and F, F and G is 0.1, 0.2, 0.2, 0.5, 0.4, 0.5, 0.1, 0.1, 0.1, 0.3 and 0.4,

respectively. The lysine composition is 0.1, 0.2, 0.3, 0.4, 0.5, 0.6 and 0.7 respectively, and only use the composition as an example to demonstrate the calculation of various network topological features.

1. According to the equation (1) and lysine composition, when L = 1 and L= 2, *APVW* feature of protein A can be calculated:

$$APVW_{A,1}(\text{Lys}) = \frac{\frac{v_A(\text{Lys})\times v_B(\text{Lys})}{1!}+\frac{v_A(\text{Lys})\times v_C(\text{Lys})}{1!}+\frac{v_A(\text{Lys})\times v_D(\text{Lys})}{1!}}{3}$$

$$= \frac{\frac{0.1\times 0.2}{1}+\frac{0.1\times 0.3}{1}+\frac{0.1\times 0.4}{1}}{3}$$

$$= \frac{3}{100}$$

$$APVW_{A,2}(\text{Lys}) = \frac{\frac{v_A(\text{Lys})\times v_B(\text{Lys})\times v_E(\text{Lys})}{2!}+\frac{v_A(\text{Lys})\times v_B(\text{Lys})\times v_F(\text{Lys})}{2!}+\frac{v_A(\text{Lys})\times v_C(\text{Lys})\times v_G(\text{Lys})}{2!}}{3}$$

$$= \frac{\frac{0.1\times 0.2\times 0.5}{2\times 1}+\frac{0.1\times 0.2\times 0.6}{2\times 1}+\frac{0.1\times 0.3\times 0.7}{2\times 1}}{3}$$

$$= \frac{43}{6000}$$

Similarly, for other amino acid composition and various protein primary structure descriptors, the corresponding *APVW* feature can also be acquired.

2. According to the equation (2), when L = 1 and L = 2, *PWPFP* feature of protein A can be obtained:

$$PWPFP_{A,1} = \frac{e^F_{A,C}}{e_{A,B}+e^F_{A,C}+e_{A,D}} = \frac{0.2}{0.1+0.2+0.3} = \frac{1}{3}$$

$$PWPFP_{A,2} = \frac{(e_{A,B}+e^F_{B,F})+(e^F_{A,C}+e^F_{C,G})}{(e_{A,B}+e_{B,E})+(e_{A,B}+e^F_{B,F})+(e^F_{A,C}+e^F_{C,G})}$$

$$= \frac{(0.1+0.1)+(0.2+0.1)}{(0.1+0.5)+(0.1+0.1)+(0.2+0.1)}$$

$$= \frac{5}{11}$$

3. When L = 1, there are 3 proteins such as B, C, D which can interact with protein A. Only protein C has a given function, therefore, N^F = {C} and $|N^F|$ = 1. The path distance between A and E, A and F as well as A and G is 2, and protein F and G have

given functions, therefore, N^F = {F, G} and $|N^F|$ = 2 when L = 2. Where, N^F indicates set of proteins with a given function and the distances between these proteins and protein A are equal to L. $|N^F|$ means the number of proteins in the set. Based on the equation (3), *APWPF* feature of protein A can be computed when L = 1 and L = 2:

$$APWPP_{A,1}=\frac{e^F_{A,C}}{1}=\frac{0.2}{1}=\frac{1}{5}$$

$$APWPP_{A,2}=\frac{(e_{A,B}+e^F_{B,F})+(e^F_{A,C}+e^F_{C,G})}{2}=\frac{(0.1+0.1)+(0.2+0.1)}{2}=\frac{1}{4}$$

4. For protein A, N = {B, C, D} when L = 1 and E_N = 3 because there are 3 edges: B-C, B-D and C-D. N^F = {C}, E^F_N= 0 because edge does not exist between C and itself. Where, N is the set of proteins with path distance L for protein A, and E_N denotes the number of edge between any two proteins in set N. E^F_N is the number of edge between any two protein in set N^F. Therefore,

$$PINPFP_{A,1}=\frac{E^F_N}{E_N}=\frac{0}{3}=0$$

When L = 2, N = {E, F, G}, E_N = 2 because there are 2 edges: E-F and F-G. N^F = {F, G}, E^F_N= 1 because there is an edge: F-G. Therefore,

$$PINPFP_{A,2}=\frac{E^F_N}{E_N}=\frac{1}{2}$$

5. The degree value of protein C is 4 because it interacts with protein A, B, D and G. The degree value of protein F is 3 because there are interactions between F and B, E, G. The degree value of protein G is 2, similarly, because interactions exist between G and C, F. Based on the equation (5), *ADPF* feature can be calculated when L =1 and 2:

$$ADPF_{A,1}=\frac{\sum_{v_{C(1)}} Deg^F_{v_{C(1)}}}{|N^F|}=\frac{4}{1}=4$$

$$ADPF_{A,2}=\frac{Deg^F_{v_{F(2)}}+Deg^F_{v_{G(2)}}}{|N^F|}=\frac{3+2}{2}=\frac{5}{2}$$

6. On the basis of the equation (6), *ADPWPF* can be obtained when L =1 and 2:

$$ADPWPF_{A,1}=\frac{e^F_{A,C}\times Deg^F_{v_{C(1)}}}{|N^F|}=\frac{0.2\times4}{1}=\frac{4}{5}$$

$$ADPWPF_{A,2}=\frac{\left(e_{A,B}\times Deg_{v_{B(1)}}+e^F_{B,F}\times Deg^F_{v_{F(2)}}\right)+\left(e^F_{A,C}\times Deg^F_{v_{C(1)}}+e^F_{C,G}\times Deg^F_{v_{G(2)}}\right)}{|\mathrm{N}^\mathrm{F}|}$$

$$=\frac{(0.1\times5+0.1\times3)+(0.2\times4+0.1\times2)}{2}$$

$$=\frac{9}{10}$$

7. When L = 1 and 2, the intersection set N^{IF} between N and N^F is {C} and {F, G}. Therefore, PP of protein A can be calculated by equation (7):

$$PP_{A,1}=\frac{|N^{IF}|^2}{|N|\,|N^F|}=\frac{1^2}{3\times1}=\frac{1}{3}$$

$$PP_{A,2}=\frac{|N^{IF}|^2}{|N|\,|N^F|}=\frac{2^2}{3\times2}=\frac{2}{3}$$

Figure S2 is selected as an example to demonstrate the calculation of PPL. According to the definition, when L = 2, there are 3 paths: B-A-C, B-A-D and C-A-D, in which initial and final vertexes are proteins with a specific function and middle vertex is protein A. There are 2 paths: C-E-D and D-F-G, in which initial and final vertexes are proteins with a specific function and middle vertex is not protein A. Therefore,

$$PPL_{i,L}=\frac{NP_i^F}{NP^F}=\frac{3}{2}$$

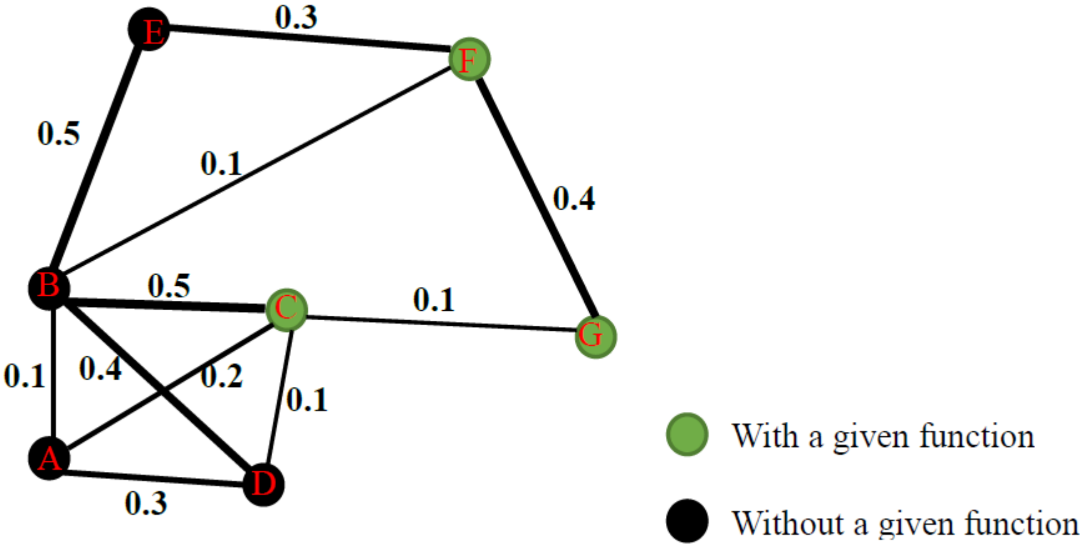

Figure S1. The topological structure of the random network. The width of edge is proportional to the corresponding interaction confidence score.

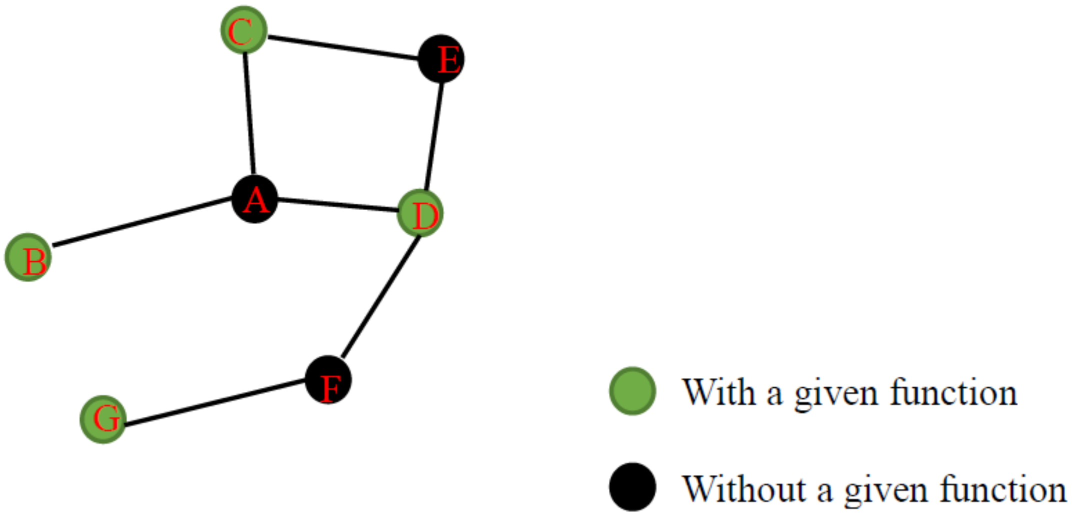

Figure S2. The topological structure of protein-protein interaction network.
